# Investigation of the effects of mTOR inhibitors rapamycin and everolimus in combination with carboplatin on canine malignant melanoma cells

**DOI:** 10.1186/s12917-021-03089-0

**Published:** 2021-12-11

**Authors:** Sarah Bernard, Andrew C. Poon, Peyton M. Tam, Anthony J. Mutsaers

**Affiliations:** 1 Department of Clinical Studies, Ontario Veterinary College, University of Guelph, Guelph, Ontario Canada; 2 Department of Biomedical Sciences, Ontario Veterinary College, University of Guelph, Guelph, Ontario Canada

**Keywords:** Canine, Cancer, Carboplatin, Dog, Everolimus, Melanoma, mTOR, Rapamycin

## Abstract

**Background:**

Malignant melanoma in dogs is considered to be largely resistant to conventional chemotherapy, although responses to carboplatin have been documented. Invasion and early metastasis are common features of certain melanoma subtypes that contribute to tumour progression despite aggressive local and systemic therapy. Upregulation of the PI3K/AKT/mTOR pathway has been observed in canine malignant melanoma and may represent a potential target for therapy. Rapamycin (sirolimus) and everolimus are commercially available small molecule inhibitors that target mTOR and therefore may have anticancer activity in canine melanoma. It was hypothesized that there is synergism between rapamycin or everolimus and platinum chemotherapy, and that combination drug treatment would inhibit target/downstream proteins involved in cell viability/proliferation and increase cell death in canine melanoma cells. It was further hypothesized that rapamycin or everolimus would impact metabolism by reducing glycolysis in these cells. Four canine melanoma cell lines were treated in vitro with rapamycin and everolimus as sole treatment or combined with carboplatin. Cell viability, apoptosis, target modulation, and glycolytic metabolism were evaluated by crystal violet colourimetric assay, Annexin V/PI flow cytometry, western blotting, and Seahorse bioanalyzer, respectively.

**Results:**

When combined with carboplatin chemotherapy, rapamycin or everolimus treatment was overall synergistic in reducing cell viability. Carboplatin-induced apoptosis was noted at 72 h after treatment compared to the vehicle control. Levels of phosphorylated mTOR were reduced by rapamycin and everolimus in all four cell lines, but activation of the downstream protein p70S6K was not consistently reduced by treatment in two of the cell lines. Both mTOR inhibitors decreased the extracellular acidification rate of canine melanoma cells, indicating reduced cancer cell glycolytic activity.

**Conclusions:**

Inhibition of mTOR by rapalogs, such as rapamycin and everolimus combined with carboplatin chemotherapy may have activity in canine melanoma. Future mechanistic investigation is warranted, including in vivo assessment of this combination therapy.

**Supplementary Information:**

The online version contains supplementary material available at 10.1186/s12917-021-03089-0.

## Background

Canine melanoma is a malignant tumour arising from melanocytes that produce pigmentation of the epithelium [1]. Sites commonly affected in dogs include the oral cavity and digits [1]. Local recurrence after surgical excision, with or without radiation therapy, followed by adjuvant chemotherapy with carboplatin was reported in 41% of patients [2], illustrating the neoplasm’s local invasiveness. Moreover, significant metastatic potential exists for oral melanoma even in cases that have no evidence of tumour spread at diagnosis. Nevertheless, lymph node and lung metastasis have been documented in 59 and 7% of cases, respectively, at the time of presentation [3]. Furthermore, a metastatic rate of 27% was observed in digital melanomas at the time of initial staging [4]. While the overall outcome is variable, most patients succumb to disease progression locally and/or distantly despite local and systemic treatment [2, 3, 5–7].

One mainstay in oncology to prevent or treat metastatic events in aggressive cancers is the use of chemotherapeutic agents. The addition of adjuvant cytotoxic chemotherapy following surgical excision does not appear to extend overall survival in canine melanoma, suggesting that this disease is largely resistant to conventional chemotherapy [6, 8]. One of the most established chemotherapeutic drugs for the treatment of canine melanoma is carboplatin, which in an early phase II study of 27 cases showed a 28% response rate when administered as a single agent [9]. Given this relatively low level of drug activity, novel strategies are needed to potentiate the effects of chemotherapy and positively impact outcome for canine melanoma.

One of the pathways shown to be aberrantly activated in both human and canine melanoma is the phosphoinositide 3-kinase/protein kinase B/mechanistic target of rapamycin (PI3K/AKT/mTOR) signalling cascade [10, 11]. This pathway is critical for cellular metabolism, growth, and survival [10]. In some cases, overactivation of this cellular signal is due to mutation or loss of the tumour suppressor gene phosphatase and tensin homolog (PTEN) [12], which has been reported in up to 59% of canine melanocytic tumours [13]. Rapamycin and derivatives like everolimus, commonly called “rapalogs”, are small molecules that target mTOR, thereby altering/inhibiting the PI3K/AKT/mTOR pathway [14]. Both drugs are well established, commercially available compounds that make them advantageous for study in dogs and humans. Exposure to rapamycin has been shown to decrease both the active form of mTOR and its active downstream protein p70-s6 kinase (p70S6K), resulting in decreased surviving fraction of canine melanoma cell lines in vitro [15].

The PI3K/AKT/mTOR signaling pathway plays an important role in cell metabolism. As previously described, reprogramming energy metabolism is considered one of the ten hallmarks of cancer [16]. One relevant metabolic change in cancer cells is the oxygen tension-insensitive transformation of glucose into lactate, called the Warburg effect [17]. While non-cancerous cells tend to use mitochondrial respiration for energy production when oxygen is available, cancer cells may preferentially use the less efficient glycolytic pathway to produce ATP [17]. One important contributor to this mechanism is hypoxia-inducible factor (HIF-1). Through mechanisms previously reviewed [18], expression of the transcription factor HIF-1 leads to aerobic glycolysis. HIF-1 has been shown to be increased by PI3K/AKT/mTOR pathway activity [19]. Therefore, upregulation of the PI3K/AKT/mTOR pathway in cancer cells can reconfigure their metabolism through inappropriate overactivation of HIF-1, leading to generation of lactate from glycolysis in an oxygen-irrespective fashion. As a result, the evaluation of glycolysis is relevant in canine melanoma cells which are known to have an active PI3K/AKT/mTOR signaling pathway.

Cellular metabolism can be measured in vitro by studying the oxygen consumption rate and extracellular acidification rate (ECAR) in the media of live cells. The Seahorse bioanalyzer is an example of a glycolysis test that utilizes three compounds: glucose, oligomycin and 2-deoxy-D-glucose (2-DG) to measure their impact on ECAR. Glucose, the first carbon substrate for glycolysis, increases ECAR, while oligomycin, an inhibitor of ATP synthase, shuts off and diverts oxidative phosphorylation to increase glycolytic output. 2-DG is a synthetic glucose analog that competitively inhibits hexokinase and glucose-6-phosphate isomerase activity to shut off glycolysis. Together, these compounds facilitate testing cellular glycolytic capacity (via ECAR) and their ability to utilize glucose under stressful conditions.

Since both rapamycin and carboplatin have demonstrated activity in canine melanoma cells and patients, respectively, as single agents, the goal of this study was to evaluate the combined effect of mTOR small molecule inhibitors with carboplatin. The study was conducted using multiple canine melanoma cell lines and cellular assays to investigate cancer cell growth/viability, apoptosis, target modulation, and glycolytic metabolism. It was hypothesized that combination treatment would inhibit the mTOR pathway, increase cellular apoptosis and decrease viability of melanoma cells. It was further hypothesized that mTOR inhibitors would reduce melanoma cell glycolytic metabolism.

## Results

### Cell viability after carboplatin, rapamycin or everolimus single-agent treatment

**Fig. 1:**
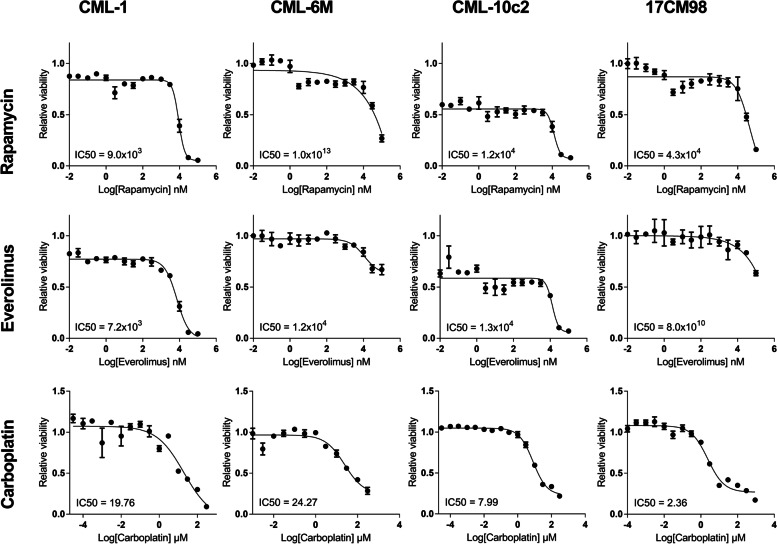
Cell viability after 72-h treatment with increasing doses of rapamycin, everolimus and carboplatin. Cell viability was measured by the crystal violet assay as described in the methods section. The error bars represent mean +/− SD of 3 experiments conducted in technical triplicate. IC50, half-maximal inhibitory concentration; mTOR, mechanistic target of rapamycin

The viability of each cell line was evaluated after single-agent treatment to guide the choice of drug doses to be utilized for subsequent combination experiments. The half-maximal inhibitory concentrations (IC50) generated using a non-linear regression model of the data points and Hill’s equation were variable among the four cell lines tested. The calculated IC50 of rapamycin ranged between 9.0 × 10^3^–1.0 × 10^13^ nM and the IC50 of everolimus ranged between 7.2 × 10^3^–1.3 × 10^4^ nM. The IC50 of carboplatin ranged between 2.3–24.2 μM depending on the cell line (Fig. 1).

### Effect of drug combination on cell viability

**Fig. 2:**
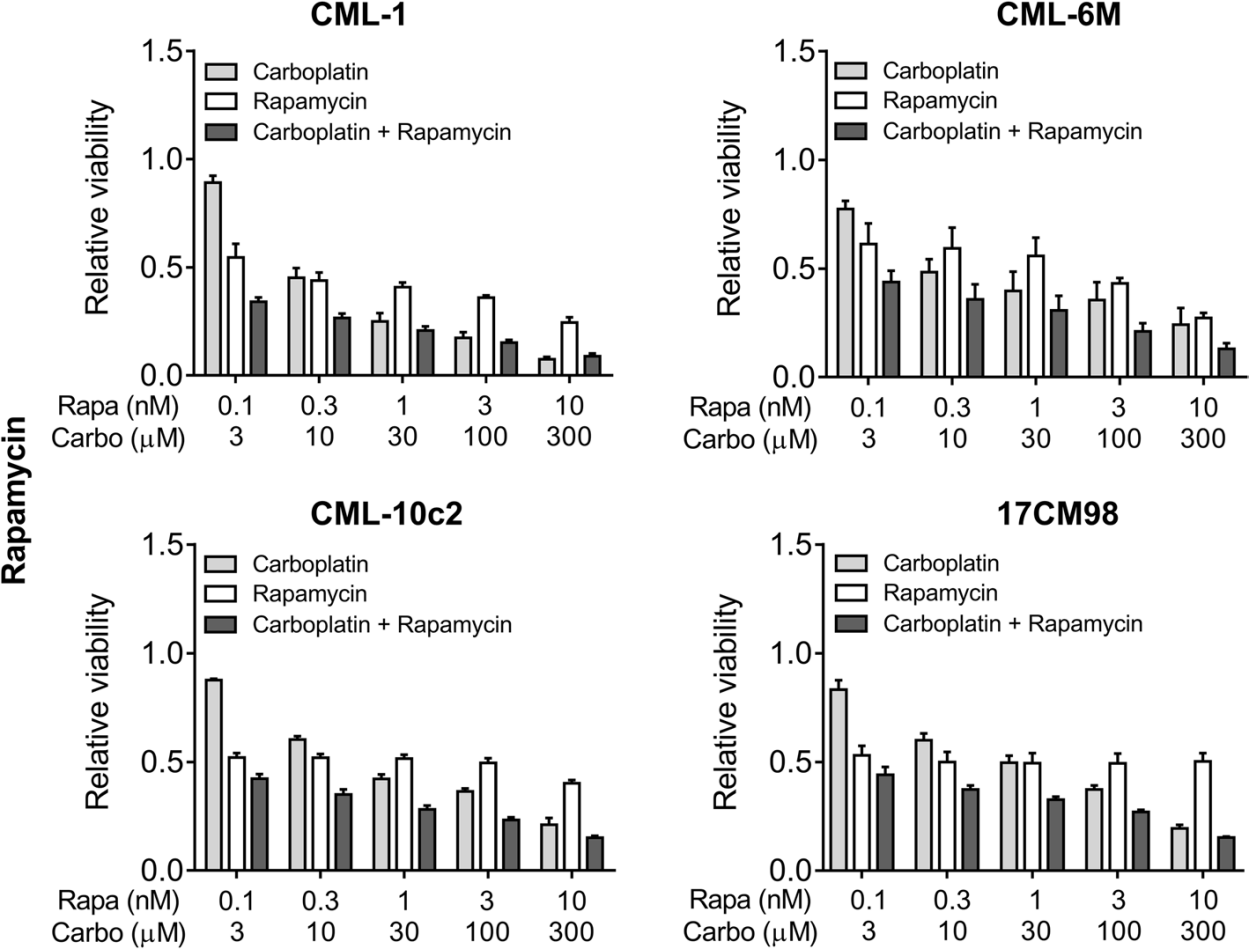
Cell viability after 72-h treatment with dose pairing of carboplatin and rapamycin. Cell viability was measured by the crystal violet assay. The error bars represent mean +/− SD of 3 experiments conducted in technical triplicate. The X-axis and the Y-axis represent dose pairings and relative viability, respectively

**Fig. 3:**
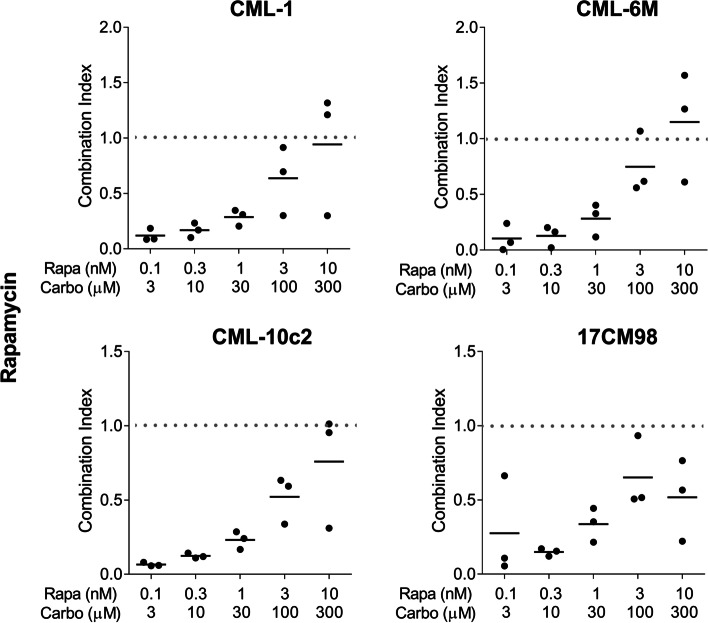
Combination Index for 72-h treatment with dose pairing of carboplatin and rapamycin. CI was calculated for all pairings. The X-axis and Y-axis represent dose pairings and CI values, respectively. The bars indicate the mean CI values. CI < 1 synergistic, CI = 1 additive, and CI > 1 antagonistic. Experiments were performed in triplicate. CI, combination index

**Fig. 4:**
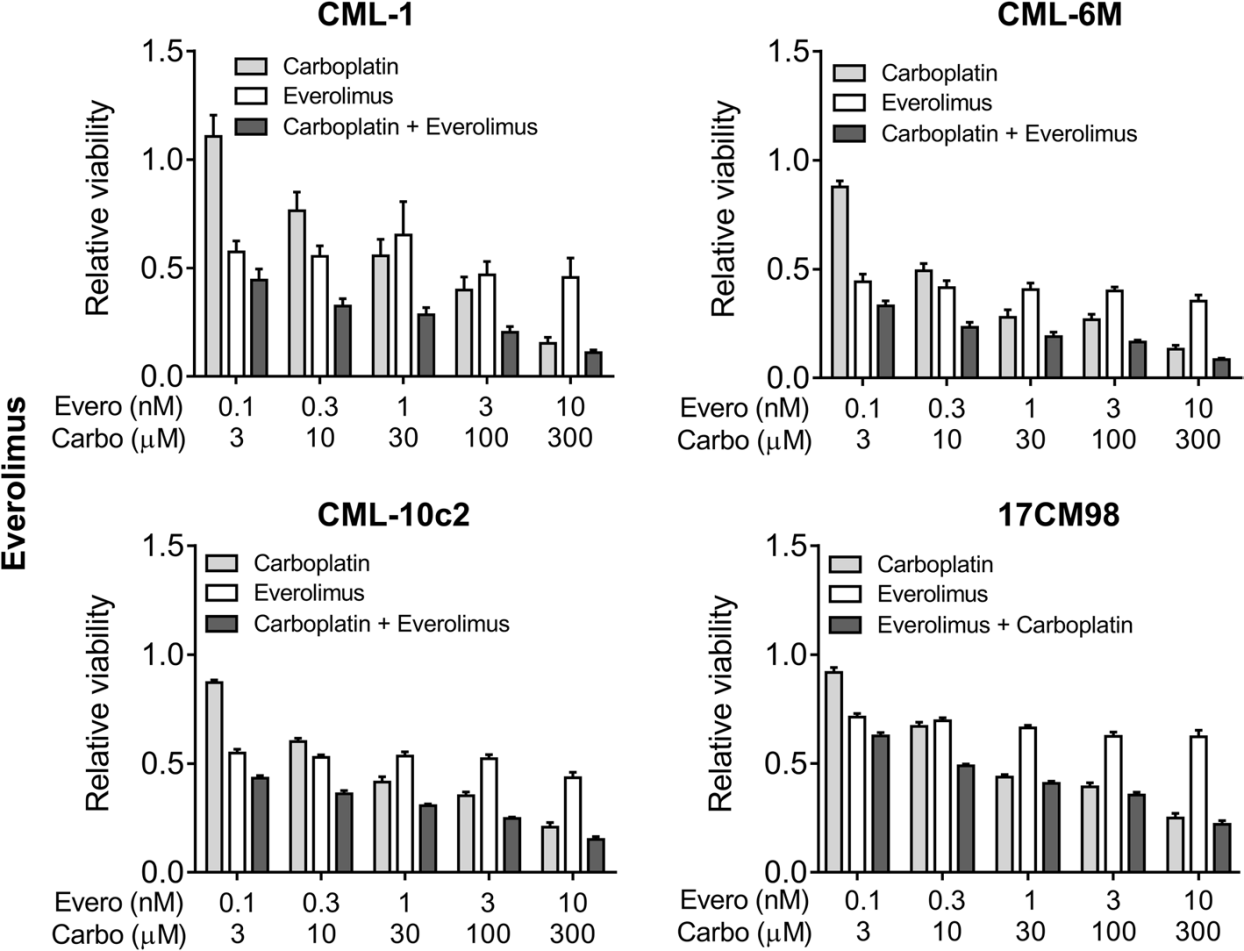
Cell viability after 72-h treatment with dose pairing of carboplatin and everolimus. Cell viability was measured by the crystal violet assay. The error bars represent mean +/− SD of 3 experiments conducted in technical triplicate. The X-axis and the Y-axis represent dose pairings and relative viability respectively

**Fig. 5:**
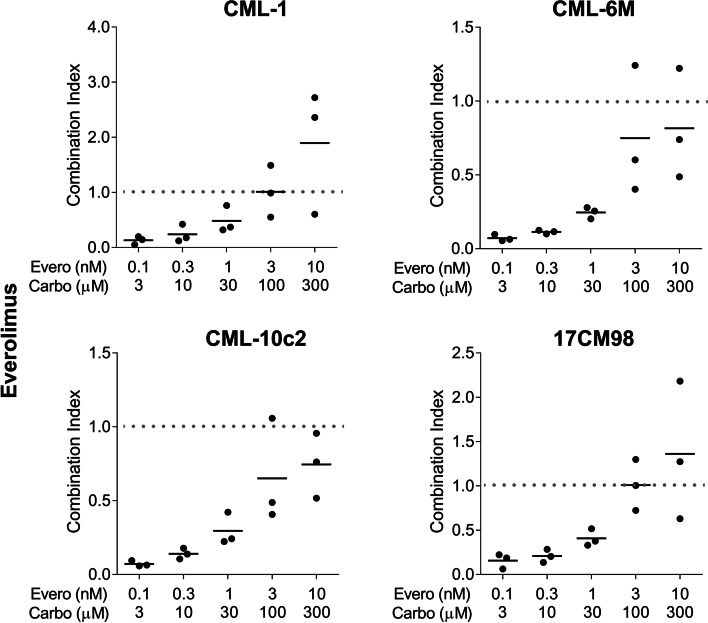
Combination Index for 72-h treatment with dose pairing of carboplatin and everolimus. CI was calculated for all dose pairings The X-axis and the Y-axis represent dose pairings and CI values respectively. The bars indicate the mean CI values. CI < 1 synergistic, CI = 1 additive, and CI > 1 antagonistic. Experiments were performed in triplicate. CI, combination index

The combination of rapamycin and carboplatin resulted in combination index (CI) values below 1, indicating drug synergy when used in combination compared to the use of either single agent. The effect was stronger when lower-dose pairings were combined. As higher dose pairings were administered, CI values were above 1 for some cell lines, indicating no superiority compared to single agent use. The mean CI value for rapamycin/carboplatin treatment for all cell lines combined was 0.14 ± 0.18 for the lowest dose pairing and 0.84 ± 0.45 for the highest dose pairing (Figs. 2 and 3). A similar trend was observed in the everolimus and carboplatin combination treated cell lines. The mean CI value for everolimus and carboplatin treatment for all cell lines combined was 0.11 ± 0.06 for the lowest dose pairing and 1.20 ± 0.78 for the highest dose pairing (Figs. 4 and 5).

### Effect of carboplatin, rapamycin and everolimus on apoptosis

**Fig. 6:**
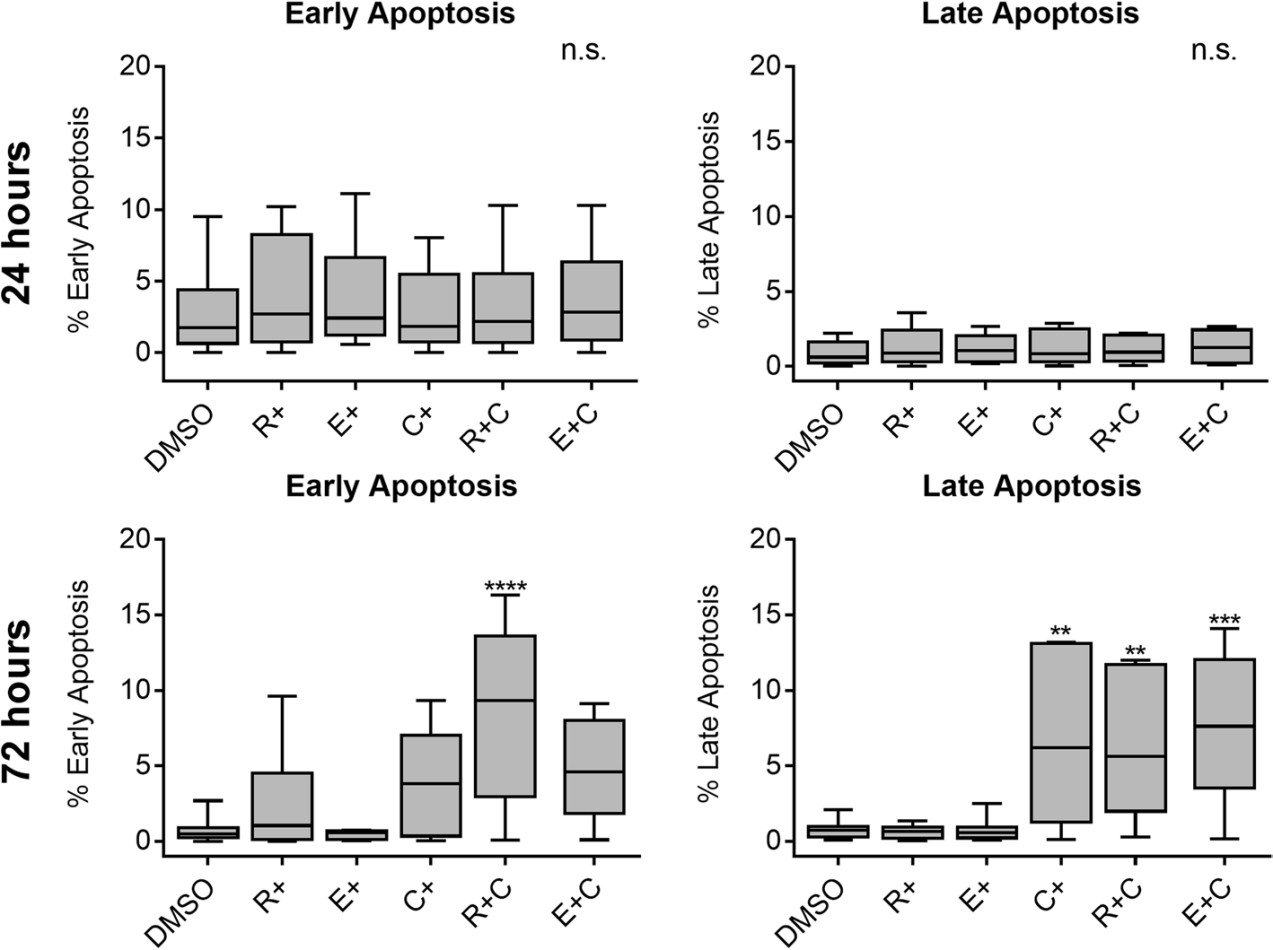
Apoptosis after treatment with carboplatin, everolimus or rapamycin. Analysis performed after 24-h and 72-h of treatment with carboplatin (15 μM), everolimus (10 nM), or rapamycin (10 nM) as a single agent or in combination. Combined analysis of all cell lines for early and late apoptosis fractions comparing single agent, combination, and vehicle treatments. Cells were stained with FITC-conjugated annexin V and PI and staining was evaluated by flow cytometry. X-axis and Y-axis represent treatments administered and % apoptosis, respectively. The error bars represent mean +/− SD of 3 experiments conducted in technical duplicate. Significance was recorded at ** *p* < .01, *** *p* < .001, **** *p* < .0001 using a one-way ANOVA, Dunnett’s multiple comparisons test, comparing treated groups to the vehicle control (DMSO). C, carboplatin; DMSO, dimethyl sulfoxide; E, everolimus; PI, propidium iodide; n.s., non-significance; R, rapamycin

To evaluate whether apoptosis was contributing to the treatment effects, annexin V and propidium iodide (PI) expression were measured by flow cytometry. Statistically significant early and late apoptosis were not observed within the first 24 h of drug treatment. However, after 72 h a statistically significant increase in early apoptosis in the rapamycin/carboplatin combination group was observed compared to the vehicle control (*p* < 0.0001). Statistically significant late apoptosis was also noted after 72 h in the single agent carboplatin (*p* < 0.01), rapamycin/carboplatin combination (*p* < 0.01), and everolimus/carboplatin combination groups (*p* < 0.001). Interestingly, subjective increases in early apoptosis were also noted in the groups treated with single agent carboplatin and everolimus/carboplatin combination, which did not reach significance (Fig. 6).

### Effect of carboplatin, rapamycin and everolimus on signalling protein expression

**Fig. 7:**
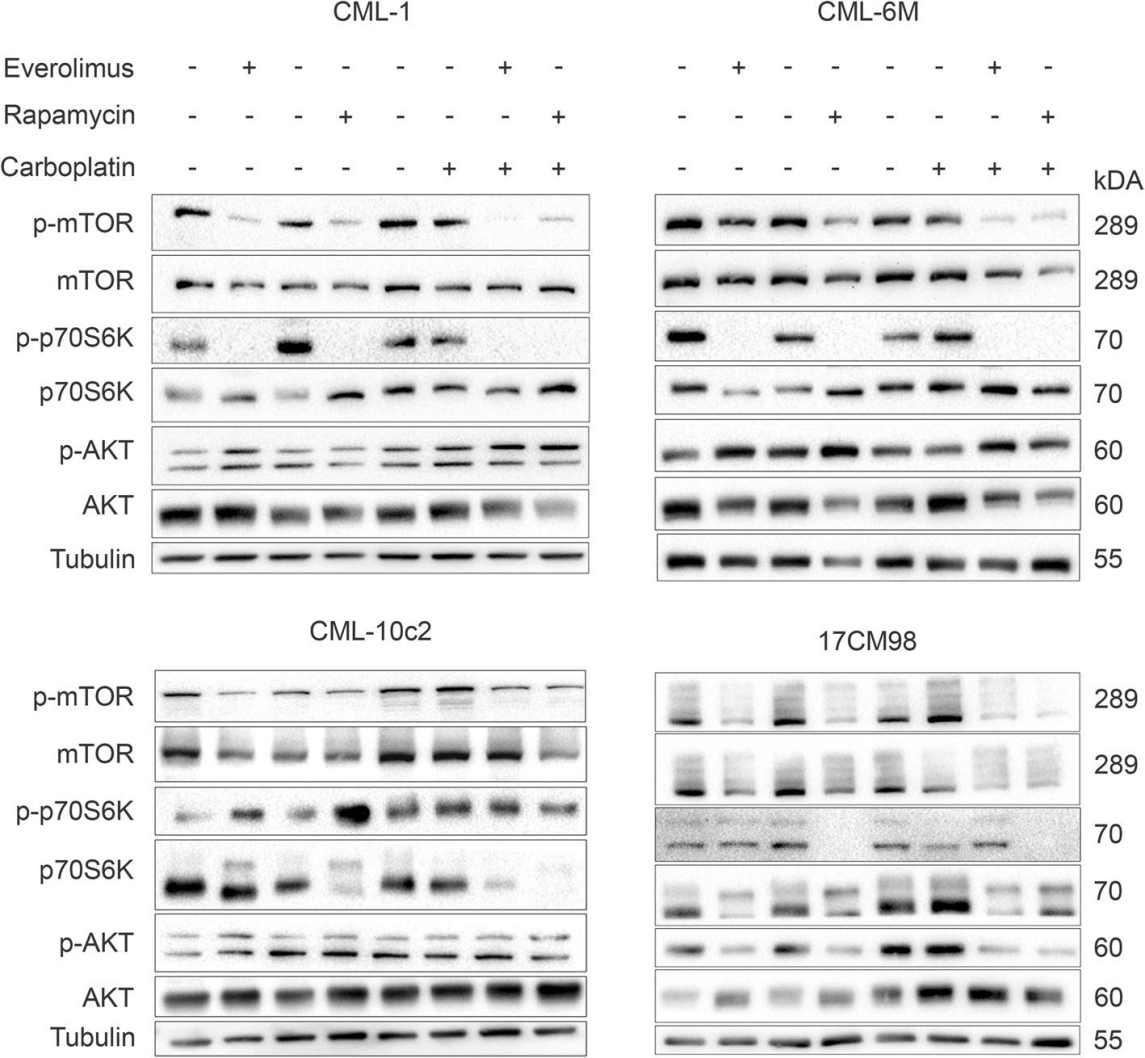
Immunoblotting of phosphorylated and native upstream and downstream proteins of mTOR in the PI3K/AKT/mTOR pathway. Analysis performed after 24-h treatment with carboplatin (15 μM), everolimus (10 nM), or rapamycin (10 nM) as single agents or in combination. The top table indicates the use of vehicle (−) or drug (+). Vehicles were DMSO for mTOR inhibitors and growth medium for carboplatin. Protein target indicated on the left with corresponding molecular weight on the right. AKT, protein kinase B; DMSO, dimethyl sulfoxide; kDa, kilodalton; mTOR, mechanistic target of rapamycin; p-AKT, phosphorylated protein kinase B; p-mTOR, phosphorylated mechanistic target of rapamycin; p-p70S6K, phosphorylated p70-s6 kinase; p70S6K, p70-s6 kinase

To confirm that rapamycin and everolimus acted through their canonical effects in melanoma cell lines in vitro, the expression of upstream and downstream proteins relevant to mTOR were qualitatively assessed. Immunoblotting revealed the presence of AKT and its phosphorylated active form in all four melanoma cell lines. AKT activation was not consistently impacted by treatment with either carboplatin or mTOR inhibitors (Fig. 7). Single-agent carboplatin also did not affect mTOR nor its downstream protein p70S6K in any cell line (Fig. 7).

Rapamycin decreased phosphorylated mechanistic target of rapamycin (p-mTOR) in all cell lines and decreased phosphorylated p70-s6 kinase (p-p70S6K) in CML-1, CML-6 M, and 17CM98, but not CML-10c2. The same impact on mTOR signalling proteins was observed when rapamycin was combined with carboplatin. (Fig. 7).

Everolimus as a single agent decreased p-mTOR in CML-1, CML-10c2, and 17CM98, but only slightly in CML-6 M. It also decreased p-p70S6K in CML-1, and CML-6 M, but not in CML-10c2 and 17CM98. When combined with carboplatin, everolimus decreased p-mTOR in all cell lines. The combination also decreased p-p70S6K in CML-1 and CML-6 M, but not in CML-10c2 and 17CM98 (Fig. 7).

Overall, the small molecule inhibitors appeared to effectively target activated mTOR in all cell lines, but its active downstream protein p70S6K was not consistently affected by treatment in CML-10c2 and 17CM98 (Fig. 7).

### Effect of rapamycin and everolimus on metabolism

**Fig. 8:**
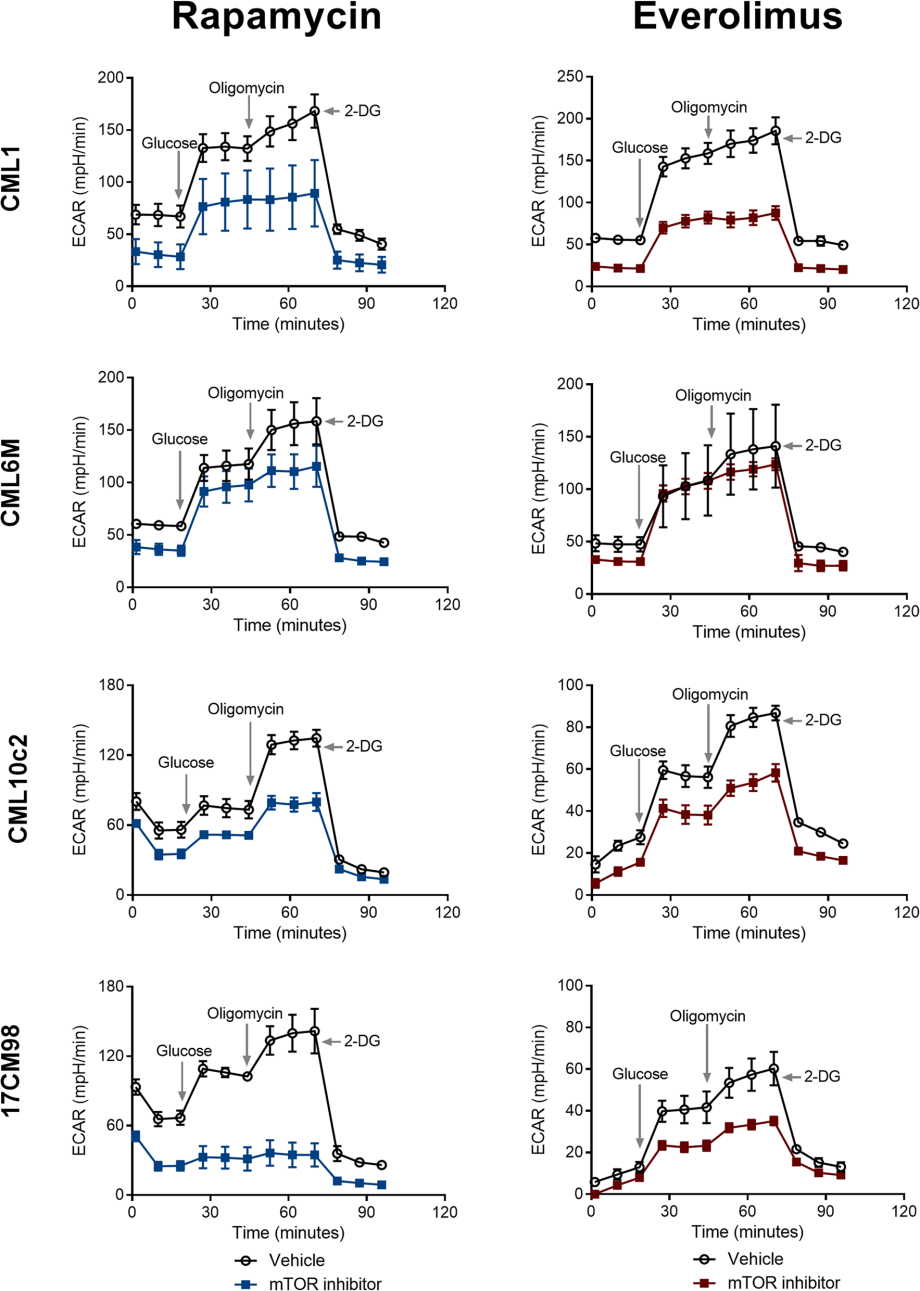
Graph representation of real-time metabolic evaluation of mTOR inhibitor treatment. Analysis performed following 24-h incubation with rapamycin (10 nM) or everolimus (10 nM). Glycolytic stress test measuring ECAR after real-time injection of glucose (10 mM), oligomycin (1 μM), and 2-deoxy-D-glucose (2-DG) (100 mM) at times indicated. X-axis and Y-axis represent time and ECAR respectively. The error bars represent mean +/− SD with a technical replicate of *N* = 10. Empty round symbols represent vehicle control. Filled squared symbols represent mTOR inhibitors. DG, Deoxy-D-glucose; ECAR, extracellular acidification rate

**Fig. 9:**
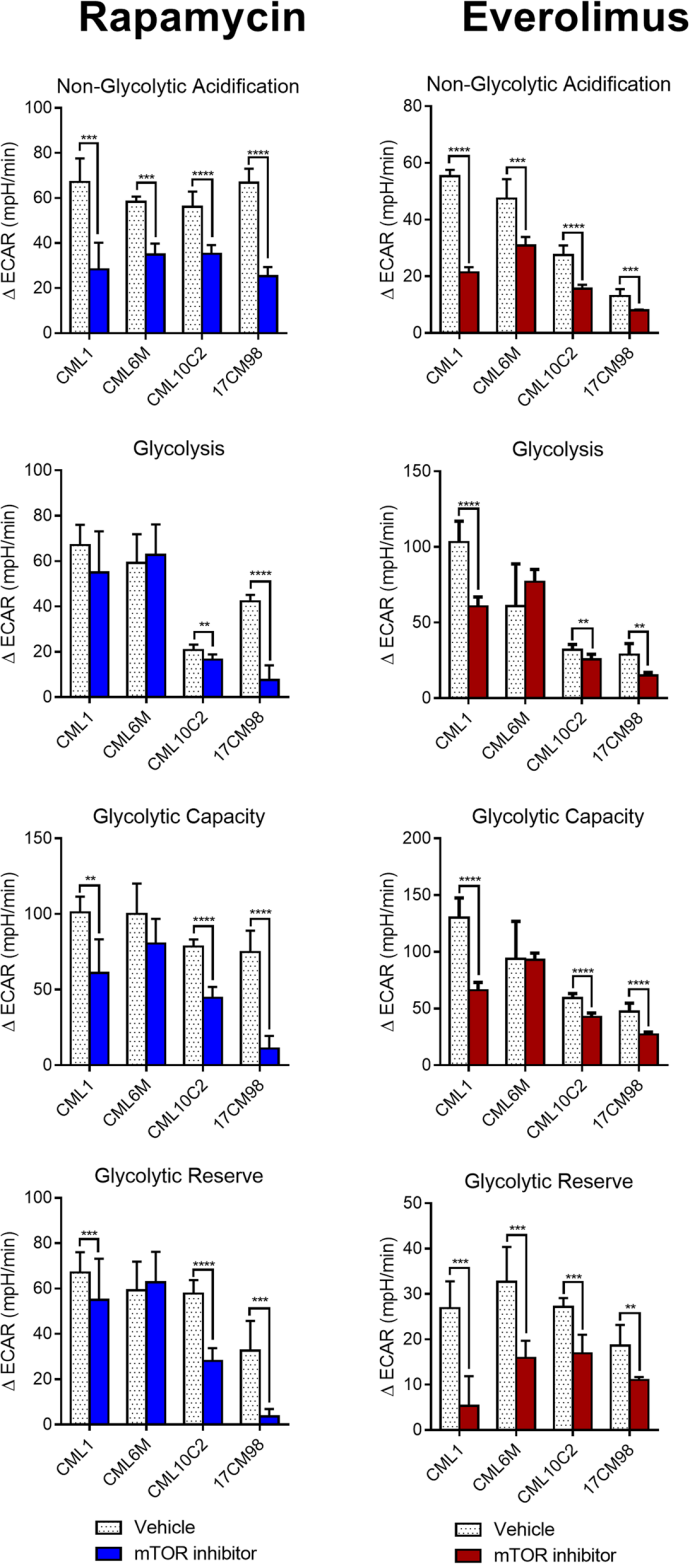
Metabolic evaluation of mTOR inhibitor treatment. Analysis performed following 24-h incubation with rapamycin (10 nM) or everolimus (10 nM). Seahorse metabolic parameters of glycolytic function measured in ECAR with drug treatment compared to vehicle for each cell line. Vehicles were DMSO. X-axis and Y-axis represent treated cell lines and ECAR respectively. Significance was recorded at ** *p* < .01, *** *p* < .001, **** *p* < .0001 using unpaired t-tests comparing treated groups to the vehicle DMSO control. The error bars represent mean +/− SD with a technical replicate of N-10. 2-DG, 2-deoxy-D-glucose; DMSO, dimethyl sulfoxide; ECAR, extracellular acidification rate

To evaluate the potential metabolic effects of rapalog treatment on canine melanoma cells, the effects on glycolysis were measured using Seahorse respirometry, to evaluate changes to the ECAR (Fig. 8). Non-glycolytic acidification represents the measure of baseline acidification in the cellular environment when analysing glycolysis. In all melanoma cell lines, a statistically significant difference in non-glycolytic acidification was detected between mTOR inhibitor treatment and control (*p* < 0.001 to *p* < 0.0001) (Fig. 9).

To investigate the effect of mTOR inhibitors on glycolysis, glucose was administered to the cells and changes in ECAR were measured. Glycolysis increases extracellular acidification when glucose is converted to pyruvate. Following glucose administration, the ECAR was statistically significantly reduced in CML-10c2 and 17CM98 cells when treated with rapamycin (*p* < 0.01 to *p* < 0.0001), and in CML-1, CML-10c2 and 17CM98 when treated with everolimus (*p* < 0.01 to *p* < 0.0001) compared to vehicle-treated control cells and the differences were statistically significant (Fig. 9).

Glycolytic capacity represents the maximum ECAR due to glycolysis after oxidative cellular respiration has been blocked by the ATPase inhibitor oligomycin, which blocks cellular respiration. Glycolytic capacity was statistically significantly reduced in CML-1, CML-10c2, and 17CM98 cells when treated with either rapamycin (*p* < 0.01 to *p* < 0.0001) or everolimus (*p* < 0.0001) compared to vehicle (Fig. 9).

Glycolytic reserve represents the change in ECAR after administration of both glucose and oligomycin, leaving glycolysis as the main ATP production mechanism. It is calculated as the difference in ECAR before and after administration of oligomycin. The glycolytic reserve was statistically significantly reduced in CML-1, CML-10c2, and 17CM98 cells treated with rapamycin (*p* < 0.001 to 0.0001), and in all four cell lines treated with everolimus (*p* < 0.01 to *p* < 0.001). Therefore, rapamycin and everolimus both decreased the glycolytic reserve of melanoma cells compared to untreated melanoma cells (Fig. 9).

Changes in ECAR measured after injection of glucose and oligomycin were confirmed to be specifically due to changes in glycolysis by injecting 2-DG, which returned ECAR to baseline levels in all cell lines (Fig. 8).

In conclusion, treatment with rapamycin and everolimus impaired the ability of canine melanoma cells to use glycolysis to produce energy, especially when cellular respiration was blocked (mimicking anaerobic conditions). These findings suggest that rapalogs could influence cellular proliferation and survival in part through the inhibition of glycolysis.

## Discussion

The aim of this study was to evaluate the activity of the PI3K/AKT/mTOR pathway in four melanoma cell lines after being co-treated with carboplatin and one of two different mTOR inhibitors. The main goal of combination therapy is to maximize therapeutic effects while minimizing toxicity from each drug. The chemotherapy drug carboplatin was selected after demonstrating clinical anti-cancer effects in just under one third of canine oral melanoma patients evaluated [9] and this drug has also been utilized in the adjuvant setting in a number of studies [2, 20]. The mTOR inhibitors were chosen as they interfere with the PI3K/AKT/mTOR pathway, a major proliferation cascade of relevance to canine melanoma. These drugs are clinically available, with rapamycin having been previously evaluated in cancer-bearing dogs.

As expected, carboplatin decreased cell viability in a dose dependent manner in all cell lines as assessed using the crystal violet method. In contrast, the mTOR inhibitor doses needed to achieve IC50 were markedly variable between cell lines and an IC50 was not always attainable, or it occurred at biologically irrelevant doses. It has been suggested that mTOR inhibitors may inhibit cell proliferation without inducing apoptosis [21, 22]. In addition, a reduction in cell viability was not observed with single agent everolimus in CML-6 M and 17CM98 which are metastatic cell lines. In line with these findings, a phase II clinical trial in human metastatic melanoma patients demonstrated low anti-tumour activity of everolimus when administered as a single agent [23]. Resistance to rapalogs as monotherapy has been documented through a variety of mechanisms [14, 22] and therefore, combining rapalogs with other drugs may improve response [22, 24].

To evaluate if there was a synergistic effect between carboplatin and rapalogs, combination index was calculated as described in the methods. In all four cell lines, carboplatin and mTOR inhibitors induced a greater synergistic effect (CI <1) at lower-dose combinations. With higher dose pairings, a similar result was not observed, indicating that one of the drugs may have masked the effect of the second. This observation is known as “antagonistic buffering” [25]. Based on these data, combining carboplatin with rapamycin or everolimus resulted in synergistic inhibition of canine melanoma cell viability.

To evaluate the effect of combining carboplatin and rapamycin or everolimus on apoptosis, cells were submitted to Annexin V/PI flow cytometry after incubation with the drugs (Fig. 6). As 24-h treatment did not lead to statistically significant apoptosis, a second time point was evaluated at 72 h post-treatment. Treatment combinations led to induction of both early and late apoptosis, whereas no effect was observed with single agent mTOR inhibitors. Early apoptosis (Annexin V+/PI-) was statistically significantly increased compared to vehicle controls in cells treated with a combination of carboplatin and rapamycin, whereas significance was approached in the cell groups treated with carboplatin only or the combination of carboplatin and everolimus. Late apoptosis (Annexin V+/PI+) was statistically significantly increased compared to vehicle controls when a treatment contained carboplatin (as a single-agent or combined with the other rapalogs). Induction of apoptosis by carboplatin is mainly caused by deoxyribonucleic acid (DNA) cross-links [26]. These results show that combination treatment of canine melanoma cells with carboplatin results in increased cell death by apoptosis.

Qualitative protein detection using western blot showed activation of the PI3K/AKT/mTOR pathway in the four canine melanoma cell lines evaluated in this study (Fig. 7). It also confirmed that treatment with rapalogs led to appropriate modulation of the active form of mTOR in all cell lines. However, the downstream protein p70S6K was not consistently inhibited in the cell lines CML-10c2 and 17CM98. This finding was consistent with previous investigations [15]. The lack of inhibition observed in some cell lines may be secondary to existing feedback loops in the pathway [14]. As previously stated, PI3K/AKT/mTOR signalling appears to be greatly involved in melanoma pathogenesis in both human and canine species [11]. Among others, this pathway allows cells to thrive through its implications in regulating metabolism, growth, and survival [27]. Blockade of this important cascade was successful in our experiments, which supports the idea that rapalog activity can inhibit an important active signalling pathway in canine melanoma.

To investigate the effects of the chemotherapeutic agents on cancer cell metabolism, Seahorse bioanalyzer analyses were performed (Figs. 8 and 9). Aerobic glycolysis, as first described as a major metabolic alteration in cancer cells by Otto Warburg, [17] contributes to melanoma’s malignant behaviour [28]. Crucial pathway alterations contribute to bioenergetic profile dysregulation in human melanoma, including B-Raf proto-oncogene serine/threonine-protein kinase (BRAF) mutations, neuroblastoma RAS viral oncogene protein (NRAS) overexpression, or deletion of PTEN [28], among others. In fact, transgenic mice overexpressing PTEN have tumor suppressive properties due, in part, to their reprogramming of oxygen consumption [29]. Overall, our results demonstrate that both rapamycin and everolimus can inhibit glycolytic rates in canine melanoma cells. Inhibiting glycolysis may impair a fundamental malignant feature of this neoplasm, which could therefore represent an attractive treatment target. However, further work is warranted to better understand the metabolic consequences of mTOR inhibition, and how these specifically relate to consequent cellular processes (i.e. cell migration, metastasis, etc.).

As deregulation of the PI3K/AKT/mTOR signalling pathway is a major component of melanoma malignancy [10, 11], much interest has been focused on its modulation in human cutaneous melanoma [10]. The role of mTOR inhibitors as potential anti-neoplastic agents has been investigated in a variety of canine cancers. Rapamycin effects have been evaluated using in vitro preclinical models of melanoma [21], mast cell tumour [30], osteosarcoma [31, 32] and prostatic carcinoma [33]. Importantly, Fowles and colleagues described the effects of rapamycin with or without the addition of mitogen-activated protein kinase (MAPK) inhibitors on canine melanoma cell lines and canine tumour isolates. Similar to our findings, the PI3K/AKT/mTOR pathway was found to be active in canine melanoma and rapamycin halted the kinase cascade [21]. In the Fowles’ study, rapamycin treatment resulted in G1 cell cycle arrest rather than cell death, which could explain the lack of apoptosis documented by mTOR inhibitors in the present study [21]. A previous study has described the clinical effects of rapamycin on canine cancer-bearing patients. The goal of that study was to establish rapamycin’s pharmacokinetics and pharmacodynamics in dogs affected by osteosarcoma [20]. In contrast to rapamycin, the effects of everolimus have been studied in vitro in canine hemangiosarcoma [34] and canine mammary carcinoma [35]. To date, there has been no report of mTOR inhibitors used clinically in canine melanoma treatment and overall, rapalog clinical trials are currently in their infancy for the treatment of cancer-bearing canine patients.

Despite both medications being mTOR inhibitors, differences between rapamycin and its derivative everolimus have been identified. Those differences have been observed clinically at the level of their half-lives, distribution, metabolism, and toxicity profiles [36]. Moreover, at the molecular level, in addition to inhibiting the mTOR complex 1 (mTORC1) like rapamycin, everolimus has superior inhibition of mTOR complex 2 (mTORC2) and also blocks extracellular signal-regulated kinase (ERK) phosphorylation, which is not the case for rapamycin [37]. In the present study, everolimus and rapamycin both repressed the mTOR signal, impacting proliferation and resulting in decreased glycolytic rates without markedly increasing cell death when administered as single agents. It is possible that clinically important differences between these drugs could be identified in vivo in dogs. However, despite the reported variation between rapamycin and everolimus, their effects on canine melanoma cell lines were generally similar.

One advantage of evaluating the drugs rapamycin and everolimus is that their safety and toxicity profiles have been well established in humans. Multiple side effects affecting a broad spectrum of systems have been reported in humans receiving rapamycin and everolimus [38]. These include, but are not limited to: skin disorders, biochemical dysregulation (such as diabetes, hyperlipidemia, hypercholesterolemia, etc.), hypertension, and haematological adverse effects [38]. Such adverse events have not been reported in canine patients receiving rapamycin at 0.1 mg/kg over a period of 10 weeks in a randomized controlled trial [39]. Likewise, in a prospective dose escalation study where the pharmacokinetics and pharmacodynamics of rapamycin at 0.01 mg/kg to 0.08 mg/kg in cancer-bearing dogs were described, a maximum tolerated dose was not attained within this dose range, as the drug was well tolerated [20]. In that study, self-limiting gastro-intestinal signs, thrombocytopenia, and a mild febrile event were recorded [20]. In dogs, everolimus has been evaluated in two studies of haematopoietic stem cell transplantation [40, 41]. While marked toxicities including graft rejection, infection and death were observed with those protocols, it is important to note that everolimus was administered with concurrent aggressive immunosuppressant therapies, such as 2 Gray total body irradiation and either mycophenolate mofetil or cyclosporine [40, 41]. Although mild side effects were previously reported, rapamycin appears to be clinically well tolerated in canine patients, illustrating rapalogs as potentially safe drugs for use in combination therapy with chemotherapeutics. Clinical studies of the combination therapy are warranted. In the present study, synergistic effects were observed between carboplatin and rapalogs. This finding suggests that, if used together, this combination could reduce the risk of adverse side effects while maximizing antineoplastic benefits in patients with melanoma.

Drug doses used in this study are clinically relevant based on previous studies. In the present study, rapamycin was administered at 10 nM in the treatment of cells for the apoptosis and metabolic assays. In a previous pharmacokinetic study of rapamycin, dogs receiving 0.08 mg/kg IM once attained a maximum serum concentration of 11.49 nM (10.5 ng/mL) [20]. In a second pharmacokinetic study, dogs receiving 0.1 mg/kg PO once had a maximum serum concentration of 9.15 nM (8.39 ng/mL) [42]. Regarding the chosen chemotherapy doses, In the present study carboplatin was administered at 15 μM in the treatment of cells for the apoptosis and metabolic assays. In a previous pharmacokinetic study, when injected at 300 mg/m2 IV, the maximum serum concentration corresponded to 270 μM and a concentration of 15 μM was achieved 2 h after injection in Beagle dogs [43]. This in vitro study therefore used drug doses that may be considered relevant in vivo.

Some limitations must be considered in this study. The first and most important limitation is the in vitro design. Neoplasms in vivo are much more biologically complex, considering their interaction with the microenvironment, mutations resulting in phenotypic changes, selective pressures, etc. Therefore, results of this preclinical study do not fully model the effects or changes that would be observed in clinical application. Furthermore, cell viability assays such as crystal violet can efficiently assess the impact of treatment in a high throughput manner in numerous cell lines with many replicates. However, clonogenic survival assays, while more labour intensive, further evaluate a cell’s ability to survive and proliferate to form colonies, and could also have been considered for this study. Although carboplatin and mTOR inhibitor use has individually been described in canine patients, the clinical toxicity resulting from their co-administration remains unknown. Further work is needed to establish the safety profile of these drugs when applied clinically in combination.

Another potential limitation is the different origins and mutational profiles of the cell lines used in this study, occasionally generating heterogeneity in the results. Indeed, rapamycin and everolimus failed to inhibit the expression p-70S6K in CML-10c2, a primary cutaneous melanoma cell line. Variability in target protein expression after treatment with mTOR inhibitors may be due to a cell line’s inherent resistance to the drugs. Since many pathways dictate cellular functions with multiple cross-talks and effectors, there may be redundancies in how mTOR controls cell survival. On the other hand, variability in the data obtained as a result of cell line diversity demonstrates that response to treatment is also expected to be variable between individual cancer patients, reenforcing the importance of evaluating more than a single cell line when conducting in vitro studies.

## Conclusion

In conclusion, rapamycin and everolimus successfully targeted the mTOR pathway in canine melanoma cells and decreased their glycolytic rate. Co-treatment at low doses with carboplatin chemotherapy resulted in synergistically decreased cell viability. Time-dependent apoptosis was observed when rapalogs were combined with carboplatin, although this effect may be mainly induced by carboplatin. As melanoma continues to represent a deadly disease in canine patients, further work to establish novel and impactful treatments is needed.

## Methods

### Cell culture

Four established canine melanoma cell lines were used: CML-1, CML-6 M, and CML-10c2 (originally described by Lauren Wolfe et al. from Auburn University), and 17CM98 from the University of Wisconsin. All cell lines were generously provided by Mike Huelsmeyer from the University of Wisconsin, Madison, Wisconsin. CML-1, CML-6 M, CML-10c2, and 17CM98 originated from an oral melanoma, a lymph node metastasis from a primary cutaneous melanoma, a primary cutaneous melanoma, and a lymph node metastasis from a primary oral melanoma, respectively [44, 45]. Cells were grown in monolayer culture in Dulbecco’s Modified Eagle Medium (DMEM) (Wisent, St-Bruno, Quebec) with 10% fetal bovine serum (FBS) (Wisent, St-Bruno, Quebec), 100 U/mL penicillin/streptomycin and 2.50 μg/mL amphotericin-B (Thermo Fisher Scientific, Waltham, Massachusetts) added to the media. Cell cultures were kept in a controlled environment at 37 °C humidified air and 5% CO_2_.

### Chemical reagents

Stock solutions of carboplatin (Accord Healthcare, Kirkland, Quebec) were maintained at 10 mg/ml, diluted in culture medium. Carboplatin (Accord Healthcare, Kirkland, Quebec) was acquired from the Ontario Veterinary College pharmacy. Stock rapamycin (Selleckchem, Houston, Texas) and everolimus (Selleckchem, Houston, Texas) were maintained 10 mM, diluted in dimethyl sulfoxide (DMSO) (Sigma-Aldrich, St. Louis, Missouri).

### Cell viability

Cell viability was evaluated using the crystal violet assay. Briefly, cells were quantified using a Countess automated cell counter (Invitrogen, Carlsbad, California) and seeded at 3 × 10^3^ cells/well in a 96-well plate. After 24 h, cells were incubated with carboplatin (3 × 10^− 5^ to 9 × 10^2^ uM), rapamycin (1 × 10^− 3^ to 1 × 10^5^ nM) or everolimus (1 × 10^− 3^ to 1 × 10^5^ nM). Colourimetric assay using crystal violet was performed after 72 h of drug incubation. All media was removed from the wells and 0.5% crystal violet biological stain (Thermo Fisher Scientific, Waltham, Massachusetts) diluted in 20% methanol (Thermo Fisher Scientific, Waltham, Massachusetts) was instilled in each well. Stain was left for 10 min then wells were gently rinsed with deionized water. Overnight drying was followed by the addition of 10% acetic acid (Thermo Fisher Scientific, Waltham, Massachusetts). Plates were rocked at 40 rpm (RPM) for 15 min and absorbance was read by a microplate reader Synergy2 (BioTek, Winooski, Vermont) at 590 nm. Carboplatin results were normalized to a vehicle control of culture medium, and rapamycin and everolimus were normalized to DMSO vehicle control. Experiments were performed in triplicate for each cell line.

### Drug combination experiments

The viability assay protocol matched those of the single-agent experiments described above. Five drug concentrations were paired, combining carboplatin (3–300 μM) with each of the mTOR small molecule inhibitors (0.1–10 nM). Experiments were performed in triplicate for each cell line.

Combination indices were utilized to evaluate potential drug interactions. The CI is a mathematical formula used to evaluate the degree of antagonism/additivity/synergism between two drugs. For each drug, the dose when used in combination is divided by the dose used alone to obtain the same effect. These ratios are calculated for each drug and added. The formula is:

$$\mathrm{CI}=\mathrm{D}1/\mathrm{Dx}1+\mathrm{D}2/\mathrm{Dx}2$$

Where D1 is the dose of a first drug given in combination, Dx1 is the dose of a first drug given as a single agent, D2 is the dose of a second drug given in combination, and Dx2 is the dose of a second drug given as a single agent. Hence, if the CI value is lower than 1, it illustrates synergism. If the CI value is equal to 1, it illustrates additivity. Finally, if the CI value is greater than 1, it illustrates antagonism [46].

### Apoptosis

Cells were quantified using a Countess automated cell counter and seeded into 6-well plates at 2.5 × 10^5^ cells per well then incubated for 24 h. Cells were treated with single-agent carboplatin (15 μM), rapamycin (10 nM), everolimus (10 nM) or a combination treatment for 24 h or 72 h. Cells were then trypsinized, collected and stained with fluorescein isothiocyanate (FITC)-conjugated annexin V and propidium iodide (PI) (eBioscience, San Diego, California) to detect apoptosis by flow cytometry. The positive control samples were obtained by heat shock treatment. An aliquot containing the corresponding cell line was immersed in 100 degrees Celsius water for 30 s to induce apoptosis. Flow cytometry was performed on a BD Accuri C6 flow cytometer (BD Biosciences, Franklin Lakes, New Jersey) with 5 × 10^4^ events collected per group. Spillover between FITC and PI (FL1 and FL3 channels) was accounted for and compensated. Experiments were run in duplicate for each cell line.

### Protein extraction

Cells were quantified using a Countess automated cell counter and were seeded into 6 cm plates at 5 × 10^5^ cells/well. Cells were incubated for 6 h in culture media and then resuspended in starvation culture media using DMEM high glucose (Wisent, St-Bruno, Quebec) with 0.1% FBS, 100 U/mL penicillin/streptomycin, and 2.50 μg/mL amphotericin-B for 18 h. Incubation was followed by administration of single agent carboplatin (15 μM), rapamycin (10 nM) or everolimus (10 nM), or a combination treatment for 24 h. Cells were preincubated for 15 min with 2 mM sodium orthovanadate (Alfa Aesar, Haverhill, Massachusetts) as a pre-treatment to preserve phosphorylated proteins. Protein was extracted in complete lysis buffer (Cell Signaling Technology, Danvers, Massachusetts) containing 20 mM Tris-HCl (pH 7.5), 150 mM NaCl, 1 mM Na_2_EDTA, 1 mM EGTA, 1% Triton-X 100, 2.5 mM sodium pyrophosphate, 1 mM β-glycerophosphate, 1 mM sodium orthovanadate, 1 μg/mL leupeptin, 1 mM PMSF, 2 μg/mL aprotinin, and 1% phosphatase inhibitor cocktail II. Plates were incubated on ice for 5 min followed by cell scraping. Collected protein was incubated for 20 min on ice prior to collection of supernatants. Protein concentration was quantified by the Bradford protein assay (Bio-Rad, Hercules, California) using a bovine serum albumin (BSA) standard curve.

### Immunoblotting

Protein lysates were loaded onto 7.5% or 10% sodium dodecyl sulfate polyacrylamide gels and run through electrophoresis using 20 μg of protein per well. A wet transfer was performed onto a polyvinylidene difluoride membrane (Bio-Rad, Hercules, California) and then blocked in 5% BSA (Wisent, St-Bruno, Quebec) in Tris-buffered saline tween 20 (TBST) (Bio-Rad, Hercules, California) for 1 h. Membranes were incubated overnight at 4 °C with the following rabbit primary antibodies: monoclonal p-mTOR #5536S, monoclonal mTOR #2983S, monoclonal p-p70S6K #9234S, monoclonal p70S6K #2708S, polyclonal phosphorylated protein kinase B (p-AKT) #9271S, and monoclonal AKT #4691S (Cell Signaling Technology, Danvers, Massachusetts) which were all diluted 1:1000 in BSA. The mouse primary monoclonal antibody alpha-tubulin #T5168 (Sigma-Aldrich, Saint-Louis, Missouri) was diluted 1:5000 in BSA and used as a loading control. Secondary rabbit or mouse antibodies conjugated to horseradish peroxidase (Cell Signaling Technology, Danvers, Massachusetts) were used for chemiluminescence imaging by a ChemiDoc MP imaging system (Bio-Rad, Hercules, California).

### Metabolism and glycolysis

A Seahorse XFe24 analyser (Agilent Technologies, Santa Clara, California) was used to measure the effects of small molecule inhibitors on metabolism of the four cell lines. Cells were quantified using a Countess automated cell counter and seeded in Seahorse XFe24 24-well cell culture microplates (Part No: 100777–004) at 4 × 10^4^ cells per well and incubated for 24 h. Cells were treated with rapamycin (10 nM) and everolimus (10 nM) for 24 h. Cells were rinsed and resuspended in Seahorse XFe media (cat# 102353–100) with 2 mM L-glutamine (Thermo Fisher Scientific, Waltham, Massachusetts) prior to performing a glycolytic stress test as per the manufacturer’s protocol. Glucose (10 mM), oligomycin (1 μM), and 2-DG (100 mM) obtained from the Seahorse XFe24 Extracellular Flux Assay Kit (Part No: 102340–100) were added into ports A, B and C, respectively, for real-time injection into the media. Blank wells were set to A1, B4, C3, and D6.

### Statistical analysis

IC50 curves were generated using a non-linear regression model and Hill’s equation in GraphPad Prism 6 (GraphPad Software Inc., La Jolla, California). CI values were calculated based on the Chou-Talalay method using CompuSyn (ComboSyn Inc., Paramus, New Jersey). Flow cytometry data was analysed using FlowJo V10 (FlowJo LLC, Ashland, Oregon). One-way ANOVA and post hoc analysis using the Dunnett multiple comparisons test was used to compare treated groups to the vehicle DMSO controls. Seahorse data was analysed with Wave 2.0 (Agilent Technologies, Santa Clara, California) to obtain ECAR for non-glycolytic acidification, glycolysis, glycolytic reserve, and glycolytic capacity analysis. An unpaired t-test and post hoc analysis using the Dunnett method were performed for analysis of Seahorse data. Statistical significance was set to *p* < 0.05 where indicated.

## Supplementary Information

**Additional file 1 **: **Supplemental Figure 1**. Uncropped western blot images.

## Data Availability

The datasets generated during and/or analyzed during the current study are available from the corresponding author on reasonable request.

## Abbreviations

2-DG: 2-deoxy-D-glucose
AKT: Protein kinase B
BRAF: B-Raf proto-oncogene serine/threonine-protein kinase
BSA: Bovine serum albumin
CI: Combination index
DNA: Deoxyribonucleic acid
DMEM: Dulbecco’s modified eagle medium
DMSO: Dimethyl sulfoxide
ECAR: Extracellular acidification rate
ERK: Extracellular signal-regulated kinase
FBS: Foetal bovine serum
FITC: Fluorescein isothiocyanate
HIF-1: Hypoxia-inducible factor
IC50: Half-maximal inhibitory concentration
MAPK: Mitogen-activated protein kinase
mTOR: Mechanistic target of rapamycin
mTORC1: mTOR complex 1
mTORC2: mTOR complex 2
NRAS: Neuroblastoma RAS viral oncogene protein
n.s.: Non-significance
p-AKT: Phosphorylated protein kinase B
p-mTOR: Phosphorylated mechanistic target of rapamycin
p-p70S6K: Phosphorylated p70-s6 kinase
p70S6K: p70-s6 kinase
PI: Propidium iodide
PI3K: Phosphoinositide 3-kinase
PTEN: Phosphatase and tensin homolog
RPM: Revolutions per minute
TBST: Tris-buffered saline tween 20
