# Supplementary material for: Investigation of the effects of mTOR inhibitors rapamycin and everolimus in combination with carboplatin on canine malignant melanoma cells
Source: BMC Vet Res. 2021 Dec 11;17:382. doi: 10.1186/s12917-021-03089-0 (PMC8665592; doi:10.1186/s12917-021-03089-0)

Supplemental Fig 1. Uncropped western blot images.

CML-1 – p-mTOR

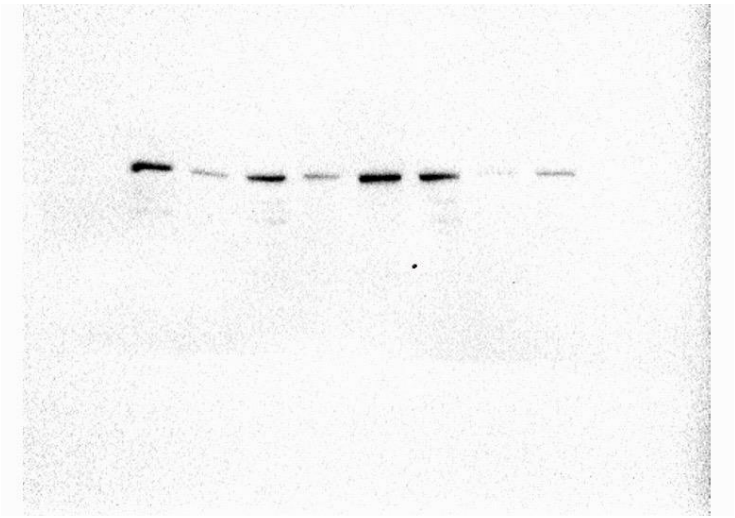

CML-1 – mTOR

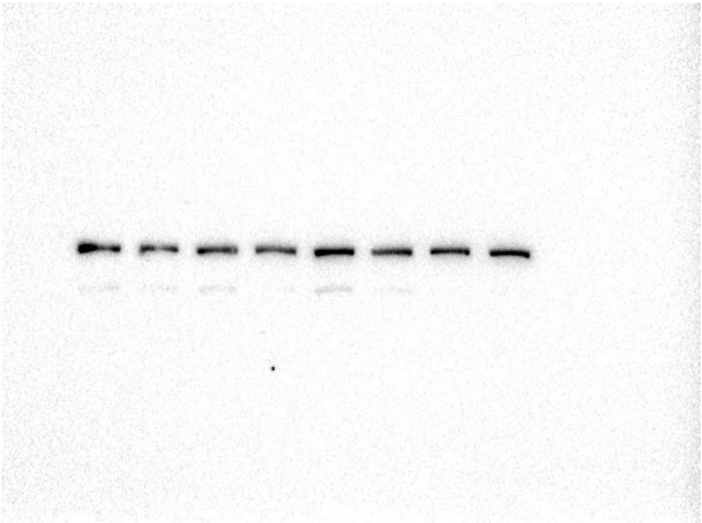

CML-1 – p-p70S6K

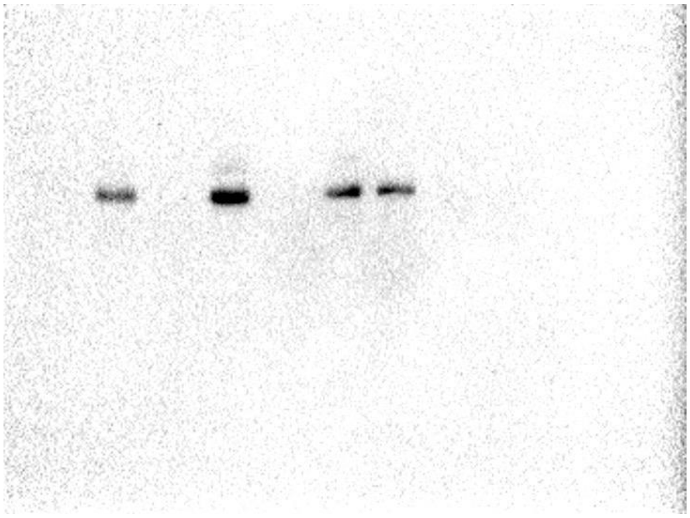

CML-1 – p70S6K

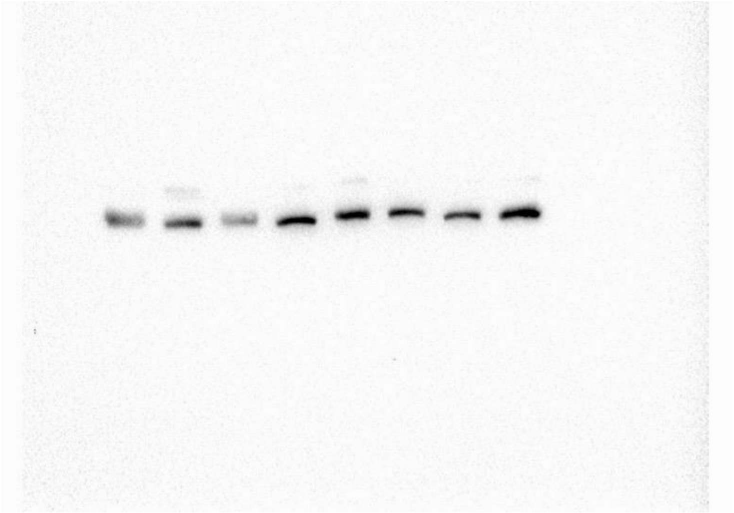

CML-1 – p-AKT

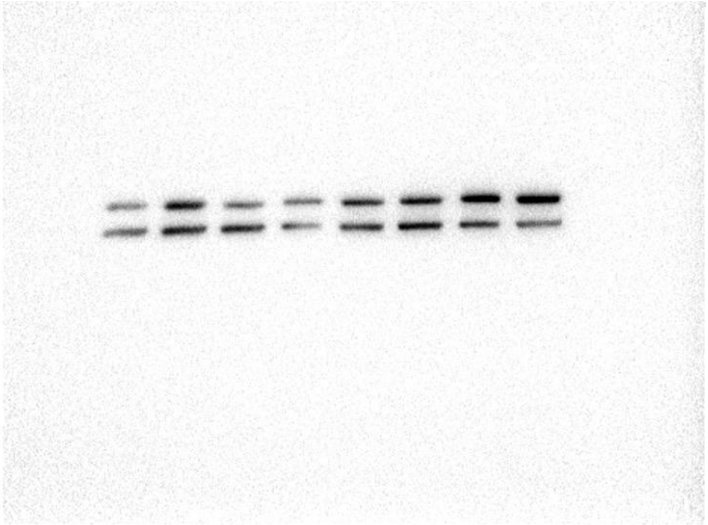

CML-1 – AKT

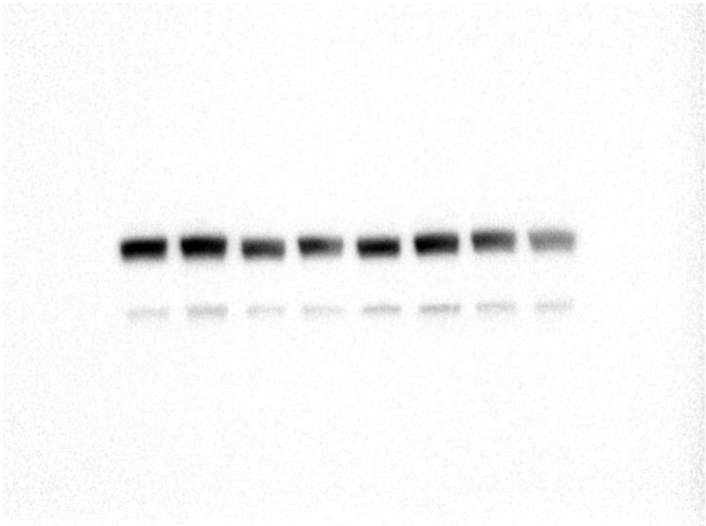

CML-1 – Tubulin

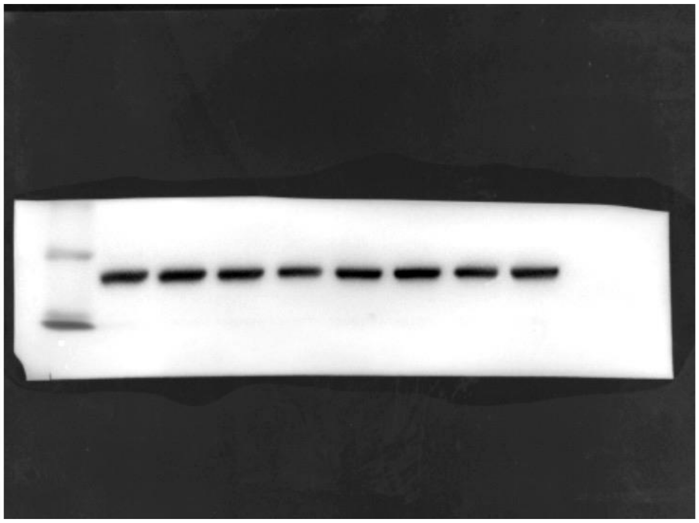

CML-6M – p-mTOR

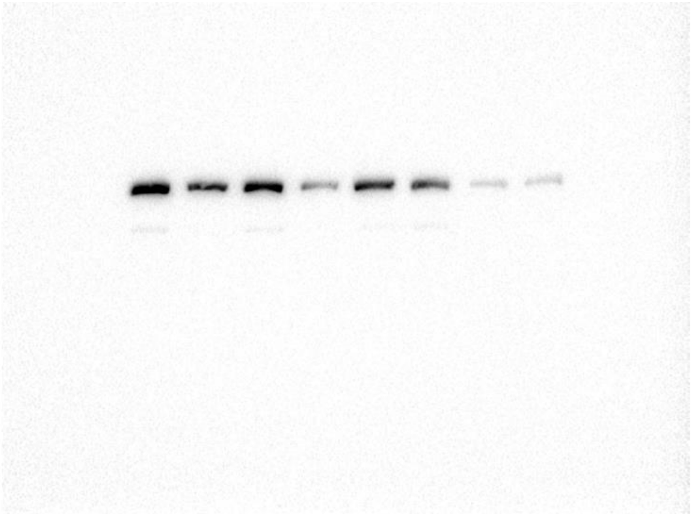

CML-6M – mTOR

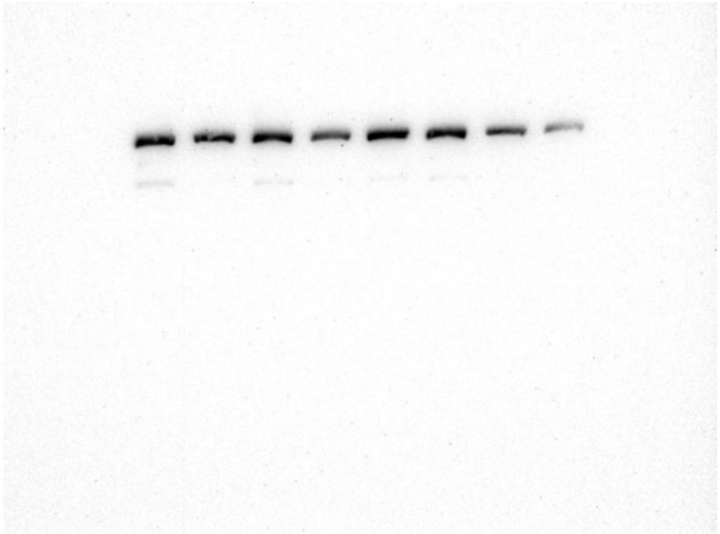

CML-6M – p-p70S6K

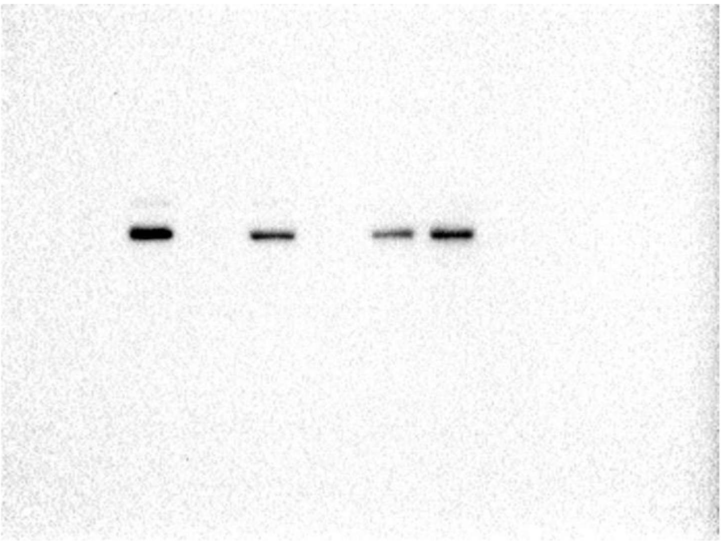

CML-6M – p70S6K

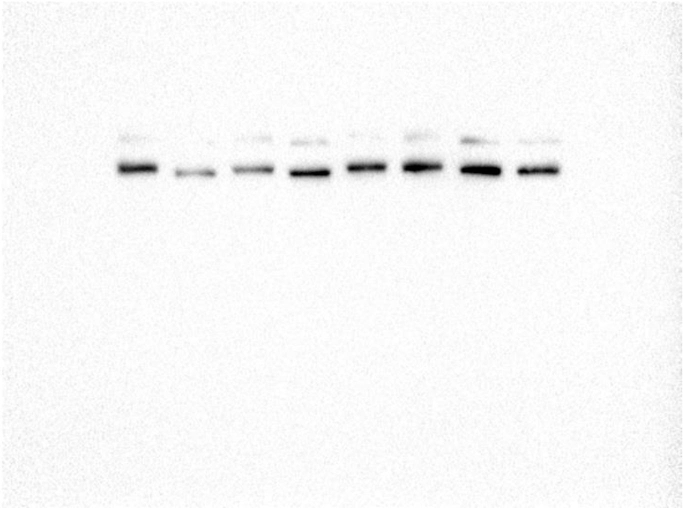

CML-6M – p-AKT

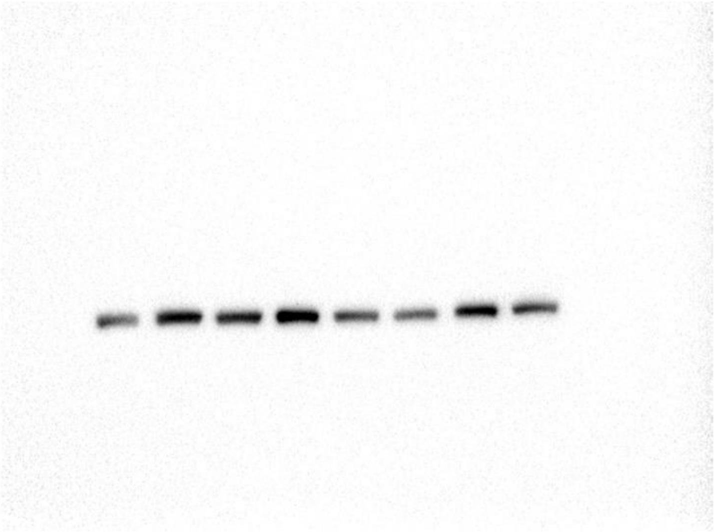

CML-6M – AKT

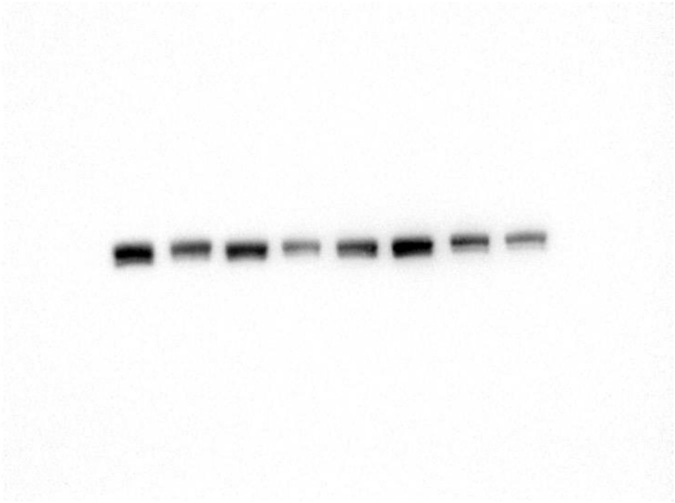

CML-6M – Tubulin

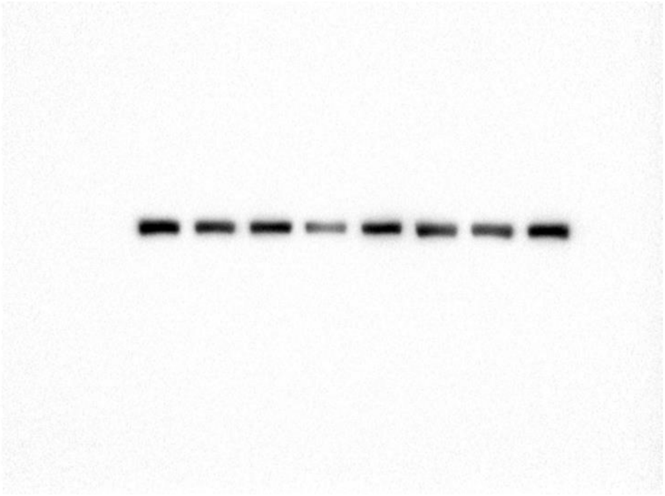

CML-10C2 – p-mTOR

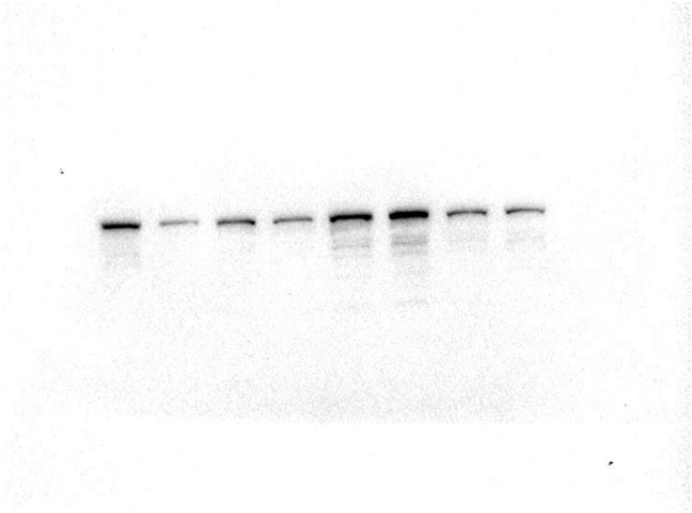

CML-10C2 – mTOR

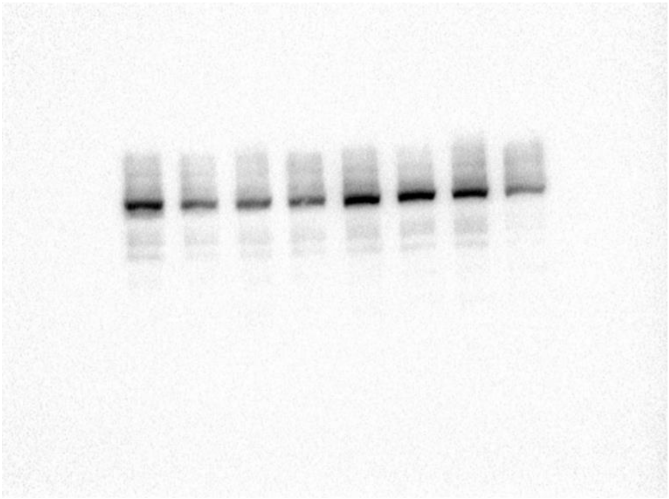

CML-10C2 – p-p70S6K

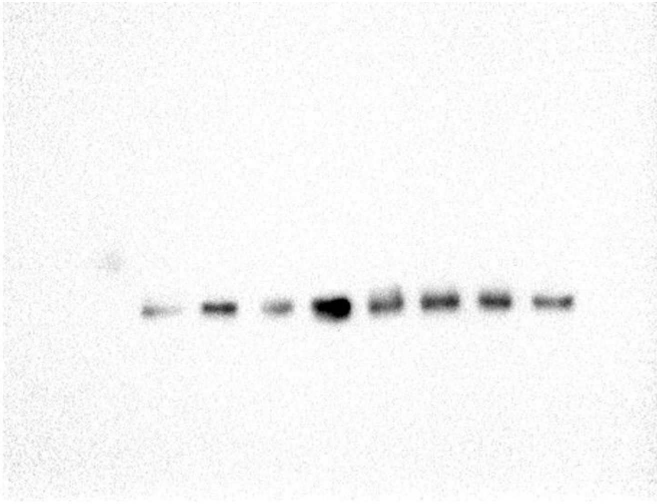

CML-10C2 – p70S6K

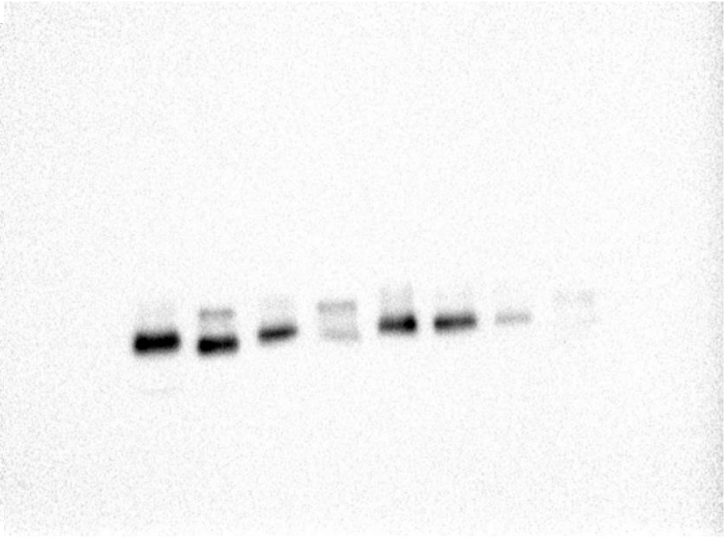

CML-10C2 – p-AKT

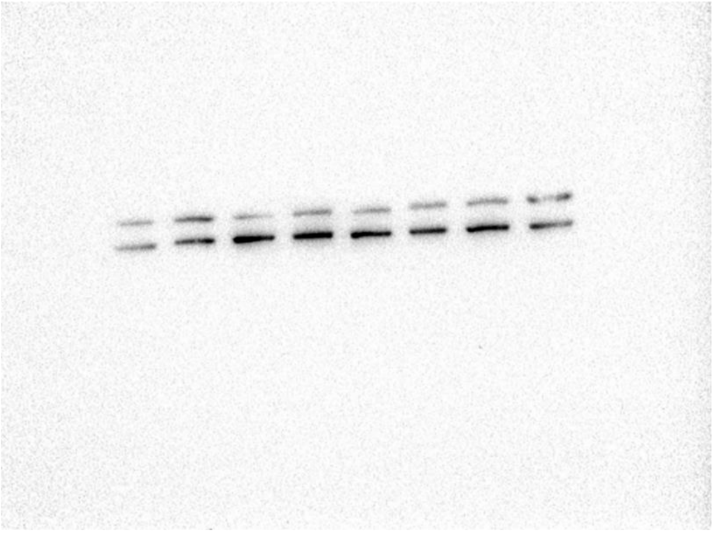

CML-10C2 – AKT

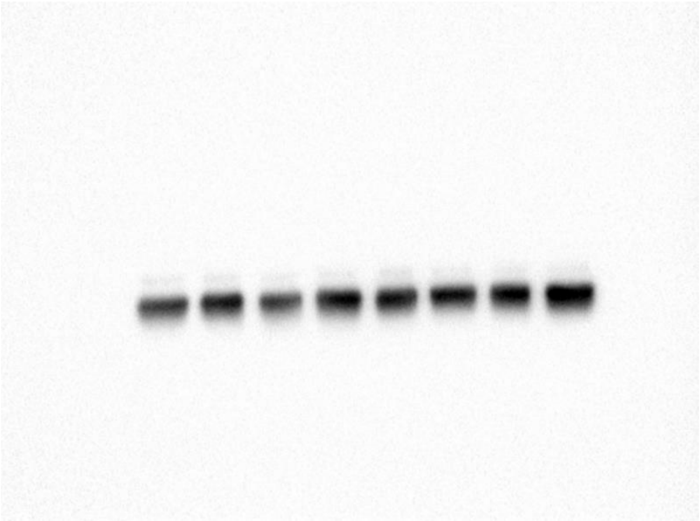

CML-10C2 – Tubulin

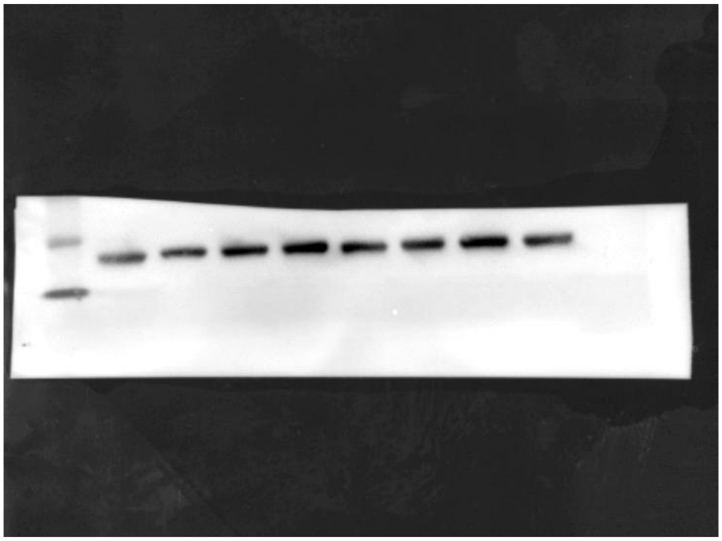

17CM98 – p-mTOR

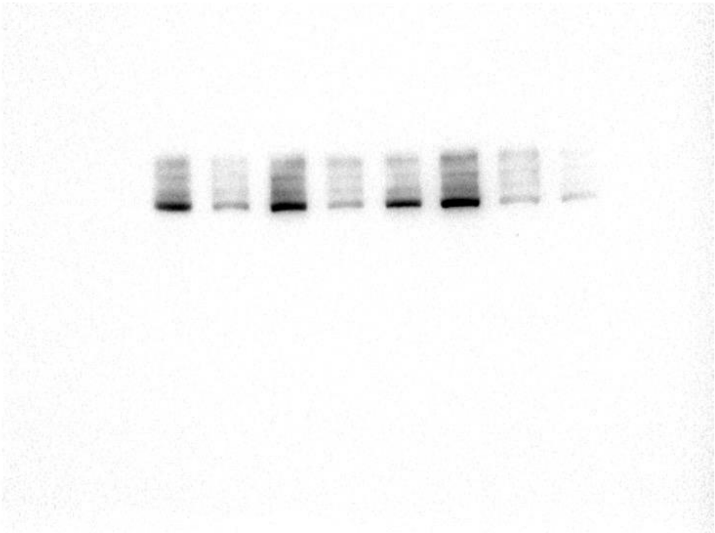

17CM98 – mTOR

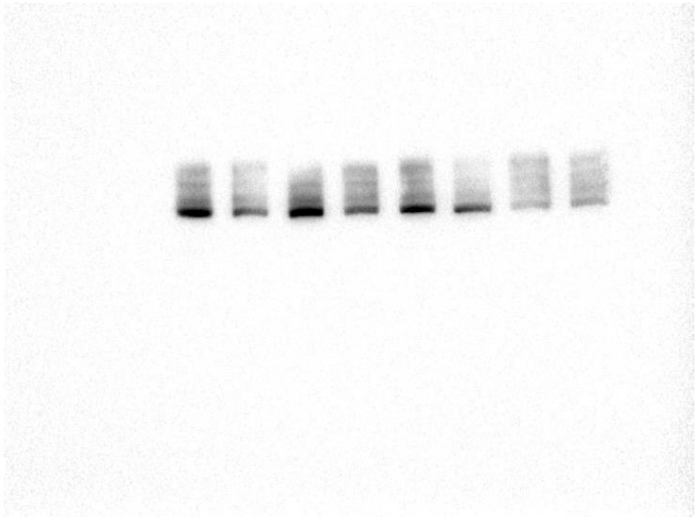

17CM98 – p-p70S6K

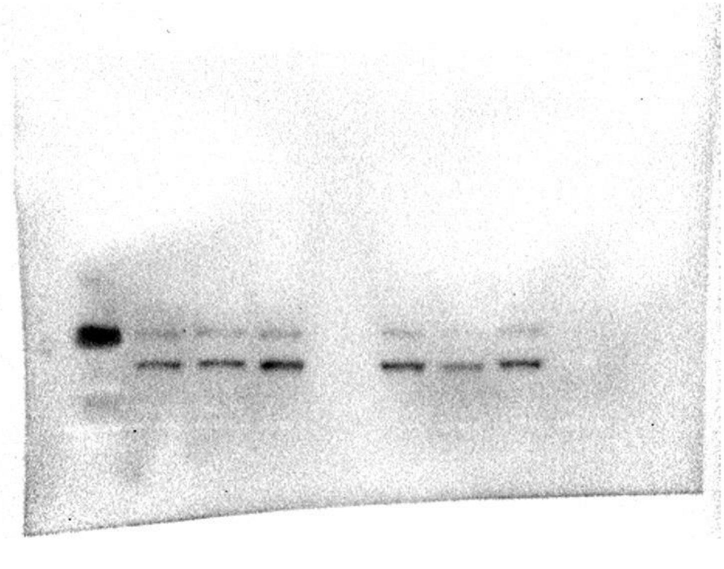

17CM98 – p70S6K

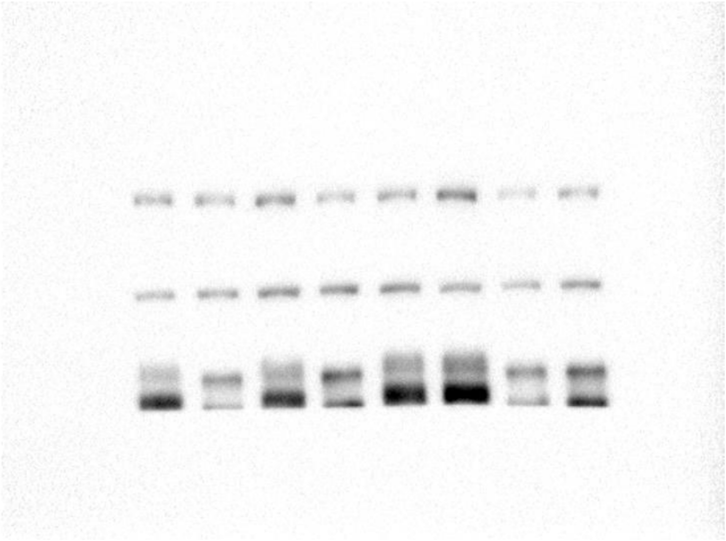

17CM98 – p-AKT

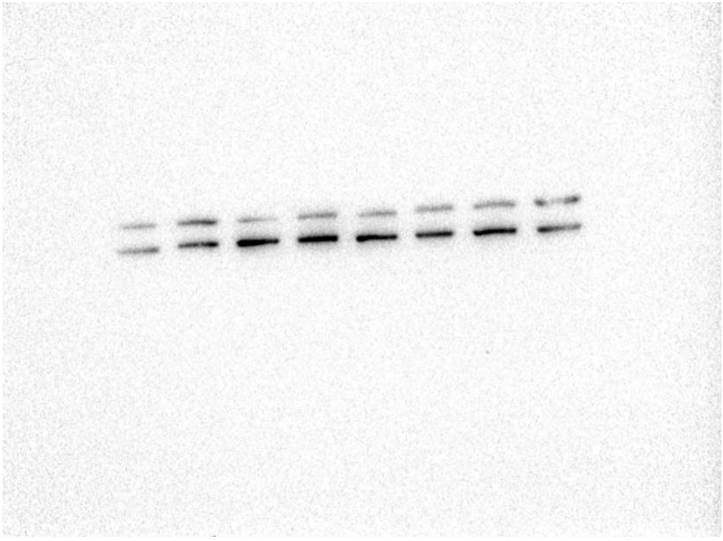

17CM98 – AKT

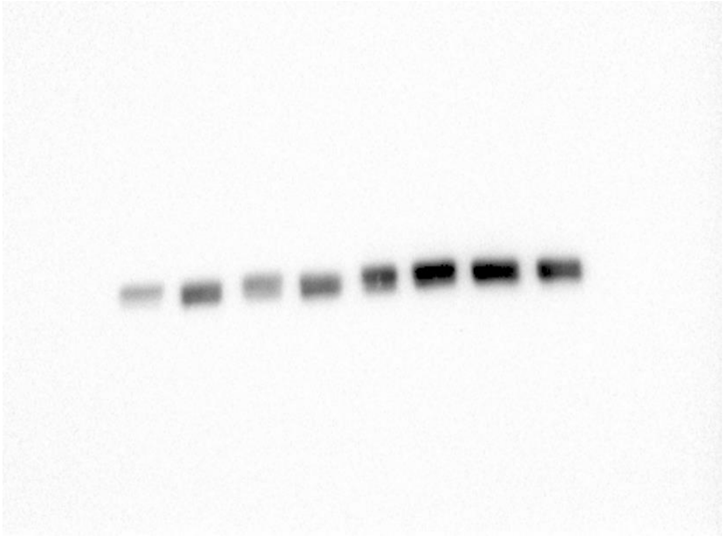

17CM98 – Tubulin

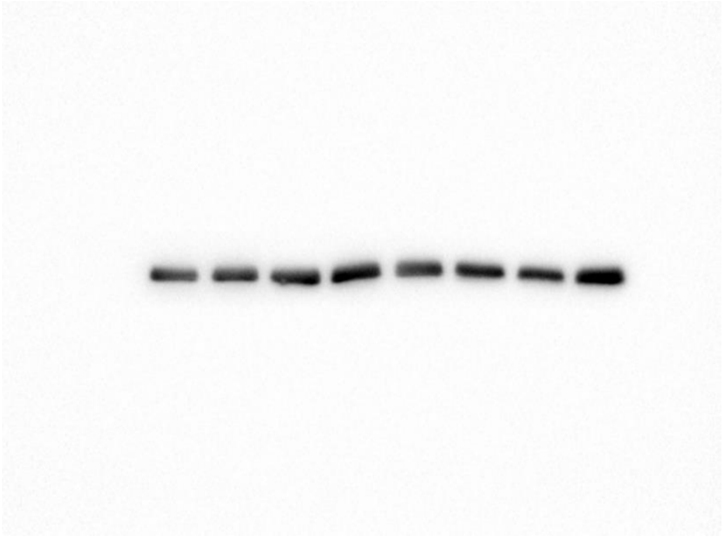

Supplement: Supplementary file 1 — Additional file 1 : Supplemental Figure 1. Uncropped western blot images. [file 12917_2021_3089_MOESM1_ESM.pdf]
